# The Role of Fucoxanthin as a Potent Nrf2 Activator via Akt/GSK-3β/Fyn Axis against Amyloid-β Peptide-Induced Oxidative Damage

**DOI:** 10.3390/antiox12030629

**Published:** 2023-03-03

**Authors:** Nayoung Lee, Kumju Youn, Jeong-Hyun Yoon, Bokyung Lee, Dong Hyun Kim, Mira Jun

**Affiliations:** 1 Department of Health Sciences, The Graduate School of Dong-A University, Busan 49315, Republic of Korea; 2 Department of Food Science and Nutrition, Dong-A University, Busan 49315, Republic of Korea; 3 Department of Pharmacology and Department of Advanced Translational Medicine, School of Medicine, Konkuk University, Seoul 05029, Republic of Korea; 4 Center for Food & Bio Innovation, Dong-A University, Busan 49315, Republic of Korea

**Keywords:** Alzheimer’s disease, amyloid-β peptide, fucoxanthin, oxidative stress, nuclear factor E2-related factor 2

## Abstract

Increasing evidence is suggesting that amyloid-β peptide (Aβ), a characteristic of Alzheimer’s disease (AD), induces oxidative stress and mitochondrial dysfunction, leading to neuronal death. This study aimed to demonstrate the antioxidant and anti-apoptotic effects of fucoxanthin, a major marine carotenoid found in brown algae, against neuronal injury caused by Aβ. Non-toxic dose range of fucoxanthin (0.1–5 µM) were selected for the neuroprotective study against Aβ_25–35_. The PC12 cells were pretreated with different concentrations of fucoxanthin for 1 h before being exposed to 10 µM Aβ_25–35_ for another 24 h. The present results showed that fucoxanthin inhibited Aβ_25-35_-induced cell death by recovering cell cycle arrest and decreasing intracellular reactive oxygen species (ROS) level. The compound enhanced mitochondrial recovery and regulated apoptosis related proteins including B-cell lymphoma 2 (Bcl-2) and Bcl-2-associated X protein (Bax) from Aβ_25-35_-induced oxidative stress. Concomitantly, fucoxanthin increased the expression of nuclear factor E2-related factor 2 (Nrf2) and its downstream phase II detoxifying enzymes including NADPH: quinone oxidoreductase-1 (NQO-1), glutamate cysteine ligase modifier subunit (GCLm), and thioredoxin reductase 1 (TrxR1), whereas it decreased the expression of cytoplasmic Kelch-like ECH-associated protein 1 (Keap1). Moreover, pretreatment of fucoxanthin reduced Fyn phosphorylation via protein kinase B (Akt)-mediated inhibition of glycogen synthase kinase-3β (GSK-3β), which increased the nuclear localization of Nrf2, suggesting that the compound enhanced Nrf2 expression by the activation of upstream kinase as well as the dissociation of the Nrf2-Keap1 complex. Further validation with a specific phosphatidylinositol 3-kinase (PI3K) inhibitor LY294002 demonstrated that the fucoxanthin-mediated Nrf2 antioxidant defense system was directly associated with the Akt/GSK-3β/Fyn signaling pathway. In silico simulation revealed that the oxygen groups of fucoxanthin participated in potent interactions with target markers in the Nrf2 signaling pathway, which may affect the biological activity of target markers. Taken together, the present results demonstrated that the preventive role of fucoxanthin on Aβ-stimulated oxidative injury and apoptosis via Akt/GSK-3β/Fyn signaling pathway. This study would provide a useful approach for potential intervention for AD prevention.

## 1. Introduction

The most common cause of dementia is Alzheimer’s disease (AD), causing a decline in memory and recognition [1]. Alzheimer’s disease is characterized by senile plaque accumulation, resulting from the extracellular deposition of amyloid-beta (Aβ) and neurofibrillary tangles, consisting mainly of hyperphosphorylated tau protein [2]. The production and accumulation of Aβ, which plays a causative role in AD pathogenesis, is generated from amyloid precursor protein (APP) through sequential cleavage by β-secretase (BACE1) and γ-secretase.

Excessive oxidative stress is one of the neurotoxic mechanisms of Aβ and is observed in the early stages of AD with markers for protein, lipid, and DNA oxidation [3]. Overproduction of reactive oxygen species (ROS) leads to oxidative stress, which accelerates Aβ accumulation and initiates a vicious cycle of mitochondrial dysfunction [4]. Furthermore, oxidative damage overwhelms the antioxidant enzyme system, leading to the disruption of the intracellular redox balance, which leads to apoptosis [5].

A growing body of evidence indicates that nuclear factor E2-related factor 2 (Nrf2) is a key regulator of the genes involved in antioxidant and detoxification responses [6]. The Nrf2 activity is negatively regulated by the repressor protein Kelch-like ECH-associated protein 1 (Keap1) that promotes Nrf2 degradation. Under oxidative stress conditions, interactions between Keap1 and Nrf2 are disrupted, and Nrf2 translocates to the nucleus where it interacts with the antioxidant response element (ARE) and induces the expression of antioxidant genes, including NADPH: quinone oxidoreductase-1 (NQO-1), glutamate cysteine ligase (GCL), thioredoxin reductase 1 (TrxR1), and other antioxidant proteins. 

Keap1-independent Nrf2 regulation involves multiple signaling cascades such as nuclear localization and nuclear export signals [7]. Among these regulatory signaling pathways, glycogen synthase kinase-3β (GSK-3β) has emerged as a point of convergence. Existing literature suggests that GSK-3β phosphorylates Nrf2, resulting in its ubiquitination and subsequent degradation [8]. Alternatively, GSK-3β may indirectly regulate Nrf2 via Fyn. Phosphorylated Fyn by GSK-3β accumulates in the nucleus and phosphorylates Nrf2, which stimulates Nrf2 nuclear export degradation to switch off the Nrf2-dependent antioxidant responses [9].

Marine algae have attracted considerable attention as a natural source of bioactive components with an important role in developing functional foods [10]. Furthermore, marine algae are crops that grow in the oceans, occupying approximately 70% of the earth’s surface, and have relatively low impacts on the environment compared to other food ingredients. Given that the world population is growing and requires more resources for climate resilience, marine algae could serve as a sustainable food source for the future [11]. 

Fucoxanthin, one of the most abundant carotenoids present in brown algae, contributes to more than 10% of total carotenoid production in nature [12]. The compound forms a complex with chlorophyll protein and plays a pivotal role in photoprotection for effective photosynthesis and light stress responses [13]. Fucoxanthin exhibits remarkable biological activities such as anti-obesity, anti-diabetes, anti-inflammatory, anticancer, and hepatoprotective effects based on its unique structure with an unusual allenic bond, 5,6-monoepoxide, which is different from that of other carotenoids, including β-carotene and astaxanthin [14]. Recent studies have shown that this compound exerts novel effects on neurodegenerative diseases [15,16,17]. Fucoxanthin inhibits BACE1, a major enzyme involved in Aβ production, and reduces Aβ fibril formation [15,16]. Additionally, fucoxanthin inhibited acetylcholinesterase (AChE), a key enzyme involved in cholinergic regulation, and decreased AChE activity and cognitive impairment in scopolamine-induced mice [17]. Moreover, the compound improved Aβ oligomer-induced memory deficits by increasing brain-derived neurotrophic factor (BDNF) expression [16]. Fucoxanthin may be a potential therapeutic alternative for AD prevention, although the underlying molecular mechanisms are still unclear. It has been speculated that the neuroprotective activity of fucoxanthin might be attributable to its dual antioxidant and anti-apoptotic properties against Aβ. Therefore, the present study was designed to discover the role of the Nrf2-mediated antioxidant response and the Akt/GSK-3β/Fyn signaling pathway in the neuroprotective effects of fucoxanthin against Aβ using in vitro and in silico approaches.

## 2. Materials and Methods

### 2.1. Sample and Aβ Preparation

Fucoxanthin (purity ≥ 95%) was purchased from Sigma–Aldrich (St. Louis, MO, USA). The Aβ_25-35_ (Genscript, Piscataway, NJ, USA) was solubilized in phosphate-buffered saline (PBS; WELGENE, Gyeongsan, Republic of Korea) at a concentration of 1 mM and incubated at 37 °C for 48 h for aggregation before use. The Aβ_25-35_ stock solution was stored at −70 °C prior to use. The solution was further diluted to 10 µM before using. The final concentration of DMSO was less than 0.01%, which did not affect the cell viability.

### 2.2. Cell Culture and Treatments

The PC12 cells were obtained from American Type Culture Collection (ATCC, Rockville, MD, USA). The cells were cultured in Roswell Park Memorial Institute (RPMI) medium containing 10% horse serum (HS), 5% fetal bovine serum (FBS), and 100 U/mL penicillin-streptomycin (all from Hyclone, Logan, UT, USA) at 37 °C and 5% CO_2_. The cells were seeded in 6-, 8-, 24-, or 96-well plates, grown for 24 h, and then replaced with a serum-free medium. The cells were pretreated with fucoxanthin at different concentrations (0.1, 1, 5, or 10 µM) or 50 µM resveratrol (Sigma–Aldrich, St. Louis, MO, USA) for 1 h before treatment with 10 µM Aβ_25-35_. For inhibitor studies, the cells were pretreated with or without 10 μM LY294002 (Sigma–Aldrich, St. Louis, MO, USA) for 30 min before treatment with or without fucoxanthin in the presence or absence of Aβ_25-35_.

### 2.3. Evaluation of Cell Viability

Cell viability was measured using the 3-(4,5-dimethylthiazol-2-yl)-2, 5-diphenyl-tetrazolium bromide (MTT; Sigma–Aldrich, St. Louis, MO, USA) assay. The MTT (5 mg/mL) stock solution was dissolved in phosphate-buffered saline (PBS) was stored at −20 °C. Cells (4 × 10^4^ cells/well) were seeded in 96-well plates and cultured for 24 h. The cells were pretreated with fucoxanthin for 1 h, followed by exposure to Aβ_25–35_ for 24 h. After incubation, the MTT solution was added to each well and incubated for another 3 h at 37 °C. The formazan crystals formed in each well were dissolved in 100 μL DMSO (Sigma–Aldrich), and absorbance was measured using a microplate spectrophotometer (Elx808, Winooski, VT, USA) at 570 nm [18].

### 2.4. Measurement of Reactive Oxygen Species (ROS) and Apoptosis

Intracellular ROS levels were detected using the redox sensitive dye CM-H_2_DCFDA (Invitrogen, Carlsbad, CA, USA). The PC12 cells (5 × 10^4^ cells/well) were seeded into 96-well plates and cultured for 24 h. The cells were pretreated with fucoxanthin for 1 h, followed by exposure to Aβ_25–35_ for 24 h. The cells were treated with 10 μL CM-H_2_DCFDA solution at 37 °C for 30 min in the dark and then washed carefully using HBSS (Gibco BRL, Grand Island, NY, USA) three times to remove non-specific staining. The fluorescence intensity of ROS was determined using a fluorescence microplate reader (Flx800, Winooski, VT, USA) with excitation and emission wavelengths of 485 and 528 nm, respectively. The cells were also imaged using a fluorescence microscope (×400, Olympus, Tokyo, Japan) [19]. 

Apoptotic cells were measured using Hoechst 33342 dye (Invitrogen). The cells (2×10^5^ cells/well) were seeded into 8-well plates and cultured for 24 h. The cells were fixed with 4% formaldehyde and stained with Hoechst 33342 dye solution at 37 °C for 15 min in the dark. Changes in the morphology of apoptotic cells were captured using a fluorescence microscope, and then cell apoptosis was counted and expressed as a percentage of the total number of cells [19].

### 2.5. Cell Cycle Assay

The cell cycle was analyzed using a Muse™ Cell Analyzer (Millipore, MA, USA). The PC12 cells (1 × 10^6^ cells/well) were seeded in 24-well plates and cultured for 24 h. The cells were pretreated with fucoxanthin for 1 h, followed by exposure to Aβ_25–35_ for 24 h. The cells were washed with PBS and fixed for 3 h at −20 °C with 70% ethanol. After washing, 200 μL Muse™ cell cycle reagent (Merck Millipore, Darmstadt, Germany) was added to each well and incubated for 30 min at room temperature in the dark [20].

### 2.6. Measurement of Mitochondrial Membrane Potential and Intracellular Free Calcium Level

Rhodamine 123 dye (Sigma–Aldrich, St. Louis, MO, USA) was used to detect the mitochondrial membrane potential (MMP). Cells (5 × 10^4^ cells/well) were seeded into 96-well plates and cultured for 24 h. Thereafter, the cells were pretreated with fucoxanthin for 1 h, followed by exposure to Aβ_25–35_ for 24 h. Then, the cells were incubated with rhodamine 123 (final concentration, 5 μM) at 37 °C for 30 min in the dark. After staining, the cells were washed with PBS, and the fluorescence intensity of MMP was measured at a 485 nm excitation wavelength and 528 nm emission wavelength (Flx800). The fluorescence signal in the cells was observed using a fluorescence microscope (× 400) [21]. 

Intracellular calcium levels were measured using Fluo-3/AM, a fluorescent dye for Ca^2+^ containing 0.02% Pluronic F-127 (Invitrogen). After treatment, the cells were rinsed for 30 min, and fluorescence intensity was detected using a fluorescence reader at a 485 nm excitation wavelength and 528 nm emission wavelength (Flx800) [21].

### 2.7. Western Blot Analysis

Cells were seeded in 6-well plates at a density of 2 × 10^6^ cells/well and treated with fucoxanthin for various durations. After treatment, cells were washed twice with cold PBS. For whole cell protein analysis, cells were lysed in extraction buffer (Cell Signaling Technology Inc., Beverly, MA, USA) containing a protease inhibitor cocktail (Tech and Innovation, Chuncheon, Korea) for 1 h on ice, and then centrifuged at 4 °C for 10 min. Cytosolic and nuclear proteins were prepared using an NE-PER Nuclear Cytoplasmic Extraction Reagent Kit (Thermo Scientific, Rockford, IL, USA). Protein concentrations were determined using the BCA method. Equal amounts of protein samples (20 µg) were separated using 8–12% SDS-PAGE and transferred to polyvinylidene fluoride (PVDF) membranes (Millipore Corporation, Bedford, MD, USA). Blotted membranes were blocked in a 5% skim milk solution in Tris-buffered saline with Tween 20 (TBST) buffer at room temperature for 2 h, and probed overnight at 4 °C with the following primary antibodies: Bcl-2, Bax, NQO1, GSK-3β, p-Fyn, Fyn, β-actin (1:2000 dilution; all from Santa Cruz Biotechnology, Dallas, TX, USA), Keap1, p-Akt, Akt, p-GSK-3β (1:2000 dilutions; all from Cell Signaling Technology, Danvers, MA, USA), GCLm (1:2000 dilution; Cusabio Technology LCC, Wuhan, China), TrxR1, Nrf2, and PCNA (1:2000 dilution; all from GeneTex Inc., San Diego, CA, USA). The membranes were then washed with TBST and incubated with horseradish peroxidase (HRP)-conjugated secondary antibodies (Bethyl Laboratories, TX, USA) at room temperature for 8–12 min. After washing with TBST, all bands were detected using Atto EZ-Capture (Tokyo, Japan) [22]. The housekeeping genes, β-actin (for whole cells) and PCNA (for nuclear fractions) were used as internal loading controls in Western blot. 

### 2.8. Reverse Transcription-Polymerase Chain Reaction (RT-PCR)

Total RNA was extracted from cultured cells using an easy-BLUETM Total RNA Extraction Kit (iNtRON Biotechnology, Seongnam, Republic of Korea). The cDNA was synthesized using TOPscript™ RT DryMIX (Enzynomics, Daejeon, Republic of Korea). The RT-PCR was performed using a Veriti™ 96-Well Thermal Cycler (Thermo Scientific, Waltham, MA, USA) with HiPi PCR PreMix (Elpis Biotech, Daejeon, Republic of Korea) [23]. The primer sequences used for RT-PCR were shown in Table 1. The housekeeping gene, GAPDH was used for the normalization of data in RT-PCR experiments.

### 2.9. In Silico Docking Simulation

The crystal structures of Nrf2 peptide for Keap1 protein (PDB ID:2FLU), Akt (PDB ID:3MVH), GSK-3β (PDB ID:1H8F), and Fyn (PDB ID:2DQ7) were obtained from the Protein Data Bank (PDB). The 3D structure of fucoxanthin was retrieved from PubChem database (CID:5281239). Simulation of the protein-binding site was performed using AutoDock Vina 1.1.2. After the docking simulation, binding poses were identified and depicted using PyMOL 2.5.0, and pharmacophore analysis was conducted using the Ligplot+ program [18].

### 2.10. Statistical Analysis

Statistical analyses were performed using SAS version 9.3 software (SAS Institute, Inc., Cary, NC, USA). Results are expressed as the mean ± standard deviation (S.D.). All data were evaluated for homogeneity and normality of variance using O’Brien’s test and Shapiro–Wilk test, respectively, and assumptions of homogeneity and normality were met. Statistical significance was evaluated using one-way analysis of variance (ANOVA), followed by Tukey’s multiple comparison test. For all comparisons, the level of significance was set at *** *p* < 0.001, ** *p* < 0.01, and * *p* < 0.05.

## 3. Results

### 3.1. Fucoxanthin Attenuated Aβ_25-35_-Mediated G0/G1 Phase Arrest and Cell Death via Reducing ROS Production

**Figure 1:**
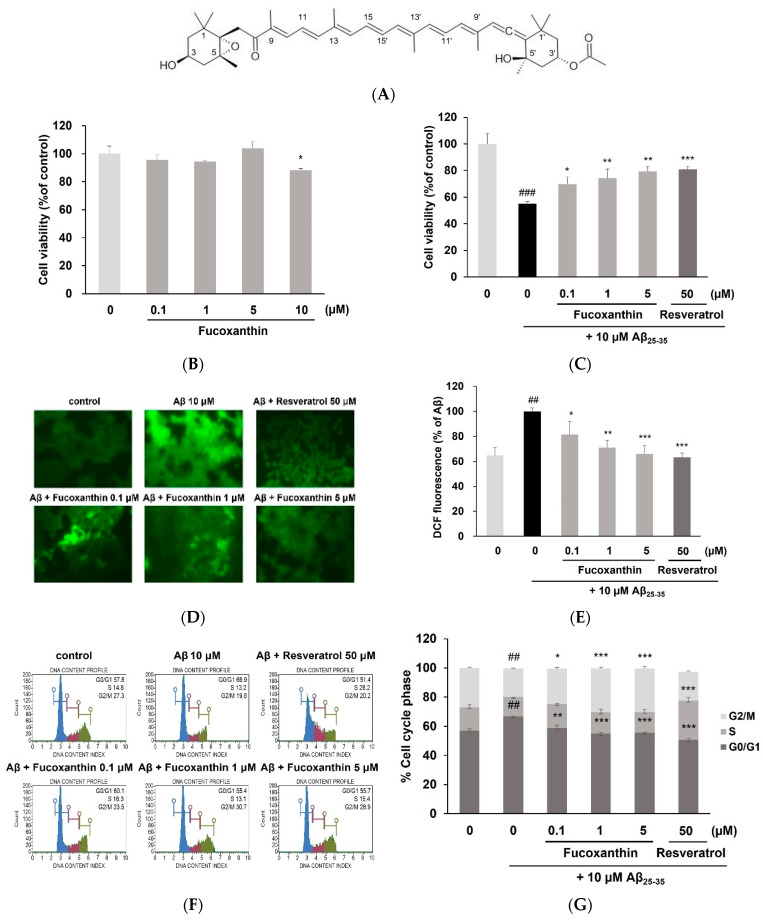
Neuroprotective effect of fucoxanthin against Aβ_25-35_-induced injury. (**A**) The chemical structure of fucoxanthin (**B**) PC12 cells incubated with various concentrations of fucoxanthin for 1 h to evaluate cell viability using MTT assay. (**C**) The cells were incubated with the sample for 1 h, followed by incubation with Aβ_25-35_ for another 24 h. After incubation, cell viability was determined by MTT assay. (**D**,**E**) Cell cycle was analyzed by flow cytometry. The cells were treated with sample for 1 h and further added with Aβ_25-35_ for 24 h. Intracellular ROS production was detected by DCF-DA using (**F**) fluorescence microscopy (400×) and (**G**) fluorescence microplate reader. Results are indicated as the mean ± S.D. and represent three independent experiments with 3 replications in each experiment. ### *p* < 0.001 and ## *p* < 0.01 compared to control group; *** *p* < 0.001, ** *p* < 0.01 and * *p* < 0.05 compared to Aβ_25-35_-treated alone.

The structure of fucoxanthin is shown in Figure 1A. As shown in Figure 1B, cell viability decreased with fucoxanthin treatment alone at 10 μM (*p* < 0.05). Accordingly, a non-toxic dose range of fucoxanthin (0.1–5 μM) was chosen for further experiments. Exposure of PC12 cells to Aβ resulted in a significant reduction (55.17 ± 1.56%, *p* < 0.001) compared with the control cells (100 ± 7.78%). However, pretreatment with fucoxanthin attenuated the Aβ-induced cell death. In particular, fucoxanthin significantly restored the cell viability at the lowest concentration (Figure 1C). Moreover, the compound showed noticeable recovery against Aβ-evoked damage at 5 μM (79.26 ± 3.81%) similar to resveratrol (80.94 ± 2.11%), which was used as a positive control. 

As there is evidence that neuronal death is intimately linked to cell division, the cell cycle of PC12 cells exposed to Aβ has been analyzed [24]. As illustrated in Figure 1D,E, Aβ_25-35_ treatment elevated the percentage of cells in the G0/G1 phase and reduced the percentage of cells in the G2/M phase in PC12 cells compared with the control. Conversely, pretreatment with fucoxanthin significantly improved the G0/G1 arrest caused by Aβ_25-35_, prolonged the G2/M phase. 

As shown in Figure 1F,G, Aβ_25-35_ significantly increased intracellular ROS levels; however, pretreatment with fucoxanthin dose-dependently eliminated ROS accumulation. In particular, fucoxanthin at 5 μM showed a strong inhibitory effect, similar to that of resveratrol (50 μM). These results showed that fucoxanthin exerted protective effect by reducing cellular ROS production, which then contributed to cell viability and cell cycle arrest in PC12 cells.

### 3.2. Fucoxanthin Enhanced Mitochondrial Recovery and Regulated Apoptosis

**Figure 2:**
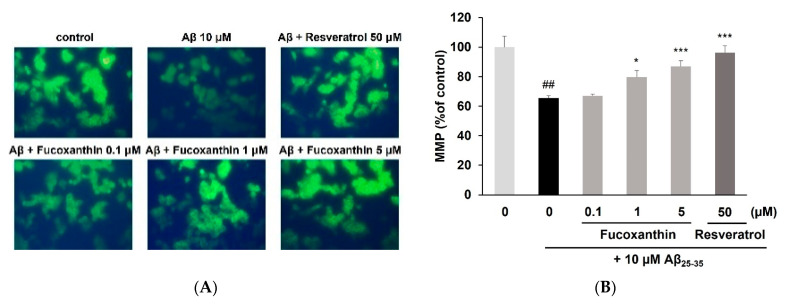

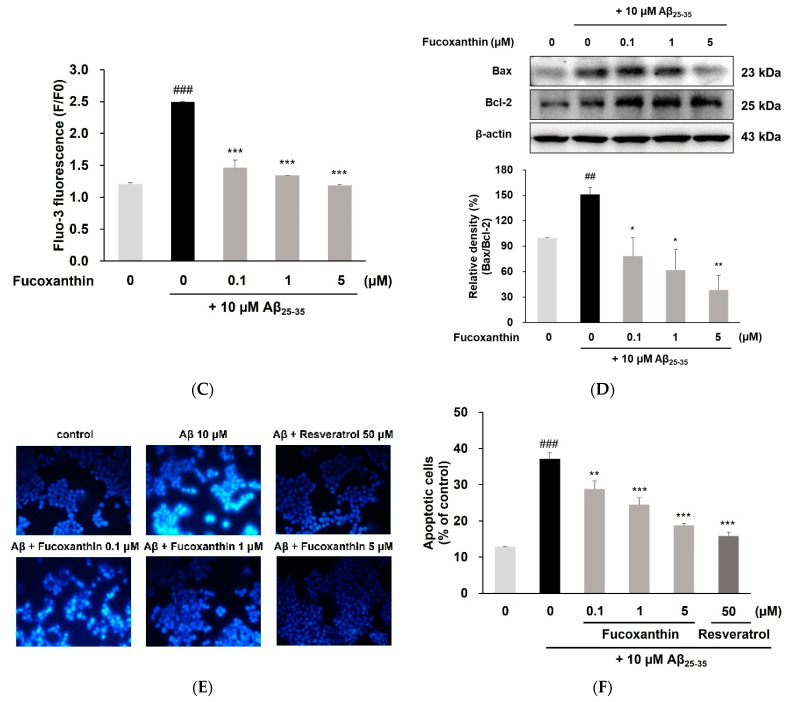
Effects of fucoxanthin on apoptosis and mitochondrial dysfunction in Aβ_25-35_-induced PC12 cells. The cells were pretreated with the indicated concentrations of fucoxanthin for 1 h and stimulated with 10 μM Aβ_25-35_ for 24 h. Mitochondrial membrane potential (MMP) observed using (**A**) fluorescence microscopy (400×) and (**B**) microplate reader. (**C**) Intracellular Ca^2+^ levels were analyzed using Fluo-3/AM. (**D**) Protein expression of Bax/Bcl ratio determined using a western blot. (**E**) Apoptotic cells were observed using fluorescence microscopy (400×). (**F**) The percentage of apoptotic cells. Results are indicated as the mean ± S.D. and represent three independent experiments with 3 replications in each experiment. ### *p* < 0.001 and ## *p* < 0.01 compared with the control groups; *** *p* < 0.001 ** *p* < 0.01 and * *p* < 0.05 compared with the Aβ_25-35_-treated alone.

As shown in Figure 2A–C, Aβ exposure resulted in the significant loss of MMP and increase of Ca^2+^ levels, whereas pretreatment of fucoxanthin prevented Aβ-caused mitochondrial dysfunction in a dose-dependent manner. Moreover, the compound significantly improved disrupting intracellular Ca^2+^ homeostasis, similar to those of the control, even at the lowest concentration. 

The results showed that fucoxanthin inhibited Aβ_25-35_-mediated up-regulation of Bax and downregulation of Bcl-2, thus decreasing the Bcl-2 to Bax ratio (Figure 2D). Moreover, in Aβ_25-35_ treated cells, the nuclear morphology appeared to be highly fluorescent condensed bodies, which are typical characteristics of apoptosis (Figure 2E). As shown in Figure 2F, the percentage of apoptotic cells in Aβ_25-35_-induced group was substantially higher than 3 times compared to the control group (*p* < 0.001). However, pretreatment with fucoxanthin significantly reduced the number of apoptotic cells at all concentrations tested, suggesting the compound attenuated mitochondrial-mediated apoptosis by Aβ via regulating MMP, Ca^2+^ overload and Bax/Bcl-2 ratio.

### 3.3. Fucoxanthin Up-Regulated Nuclear Translocation of Nrf2 and Gene Expression of Phase-II Enzyme on PC12 Cell Injury Caused by Aβ_25-35_

**Figure 3:**
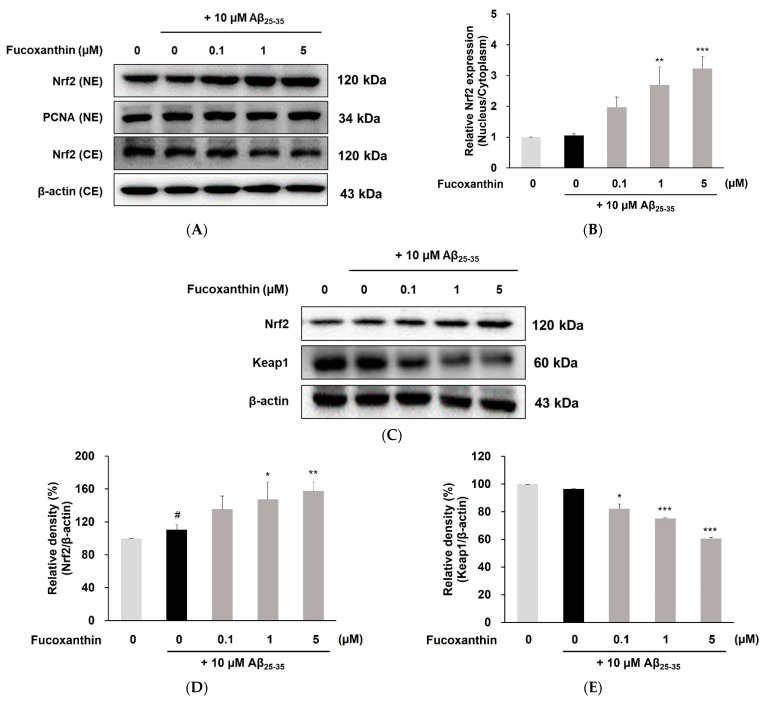

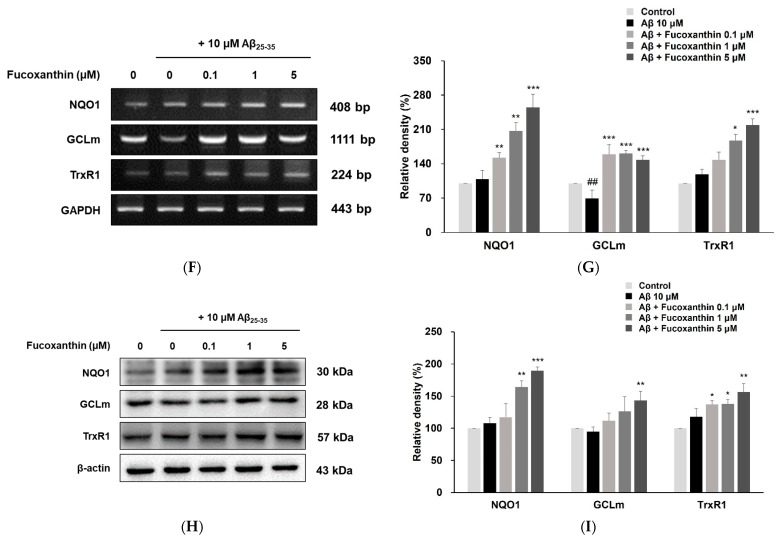
Effects of fucoxanthin on expression of Nrf2 and Keap1 in Aβ_25-35_-induced PC12 cells. The cells were pretreated with the indicated concentrations of fucoxanthin for 1 h and stimulated with 10 μM Aβ_25-35_ for 4 h. (**A**) The nuclear and cytoplasm expression of Nrf2 as measured by western blot. (**B**) Relative ratio of nuclear and cytoplasmic Nrf2. (**C**) The total Nrf2 and Keap1 expression. Quantification of (**D**) total Nrf2 and (**E**) Keap1 protein expression levels. NE, nuclear extract; CE, cytoplasmic extract. β-Actin and PCNA were used as the loading control for whole cells and nuclear fractions, respectively, in western blot assay. (**E**) Keap1 protein expression levels. NE, nuclear extract; CE, cytoplasmic extract. mRNA (**F**,**G**) and protein (**H**,**I**) levels of phase-II enzymes including NQO1, GCLm, and TrxR1. The cells were pretreated with indicated concentrations of fucoxanthin for 1 h and stimulated with 10 μM Aβ_25-35_ for 24 h. The mRNA expression levels of phase-II enzymes were determined by RT-PCR analysis. For the normalization of mRNA expression GAPDH was used as the loading control. Results are indicated as mean ± S.D. and represent three independent experiments with 3 replications in each experiment. ## *p* < 0.01 and # *p* < 0.05 compared with the control groups; *** *p* < 0.001, ** *p* < 0.01 and * *p* < 0.05 compared with the Aβ_25-35_-treated alone.

To further identify the molecular mechanisms underlying the antioxidant effects of fucoxanthin on Aβ_25–35_-induced cell injury, Nrf2, a master regulator of the antioxidant response, and its inhibitor Keap1 were investigated. Quantitative analysis of the Nrf2 nucleus/cytoplasm ratio showed that the Aβ-treated group had no effect on the nuclear accumulation of Nrf2, whereas fucoxanthin at 1 and 5 μM concentrations resulted in a more than three-fold increase in Nrf2 translocation compared with that in the control (Figure 3A,B). As shown in Figure 3C,D, exposure to 10 μM Aβ_25-35_ resulted in a significant increase in total Nrf2 expression (*p* < 0.05). Additionally, total Nrf2 protein levels were markedly upregulated by fucoxanthin in a dose-dependent manner. An inhibitory effect of fucoxanthin at all concentrations on cytoplasmic Keap1 was also observed (Figure 3C,E).

These observations showed that fucoxanthin promoted Nrf2 nuclear translocation and Nrf2 activation. Thus, we hypothesized that fucoxanthin regulates Nrf2 downstream target genes, including NQO1, GCLm, and TrxR1. As shown by PCR (Figure 3F,G) and western blot analysis (Figure 3H,I), pretreatment with fucoxanthin significantly increased the expression of NQO1, GCLm, and TrxR1 at both mRNA and protein levels. In particular, fucoxanthin at 0.1, 1, and 5 μM, resulted in approximately 1.5-, 2.0-, and 2.5-fold increases in NQO1 protein expression, respectively.

### 3.4. Fucoxanthin Modulated Akt/GSK-3β/Fyn Signaling against Aβ Neuronal Damage

**Figure 4:**
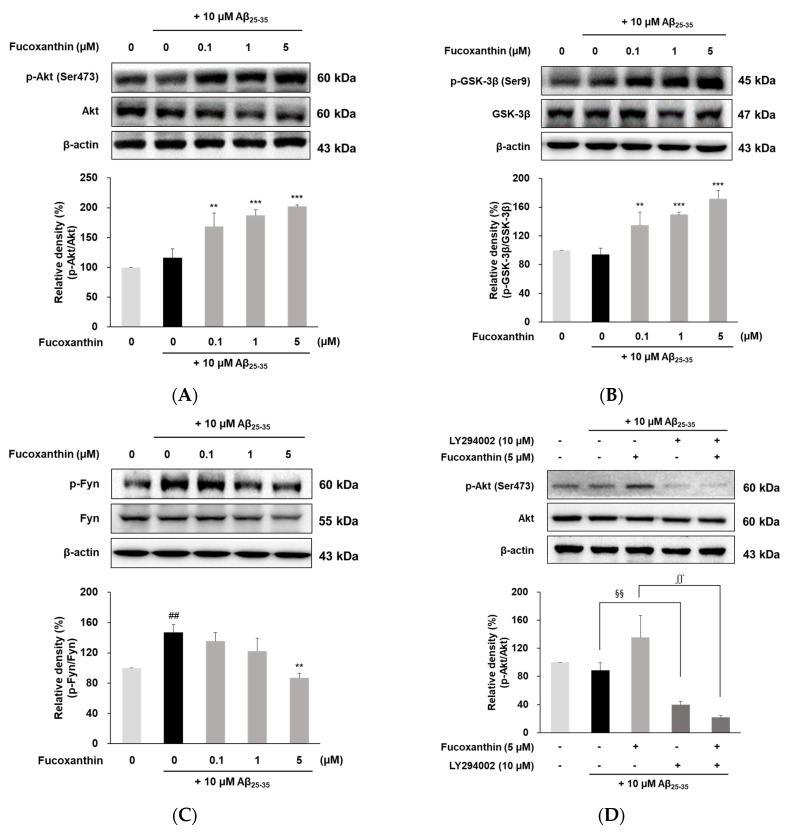

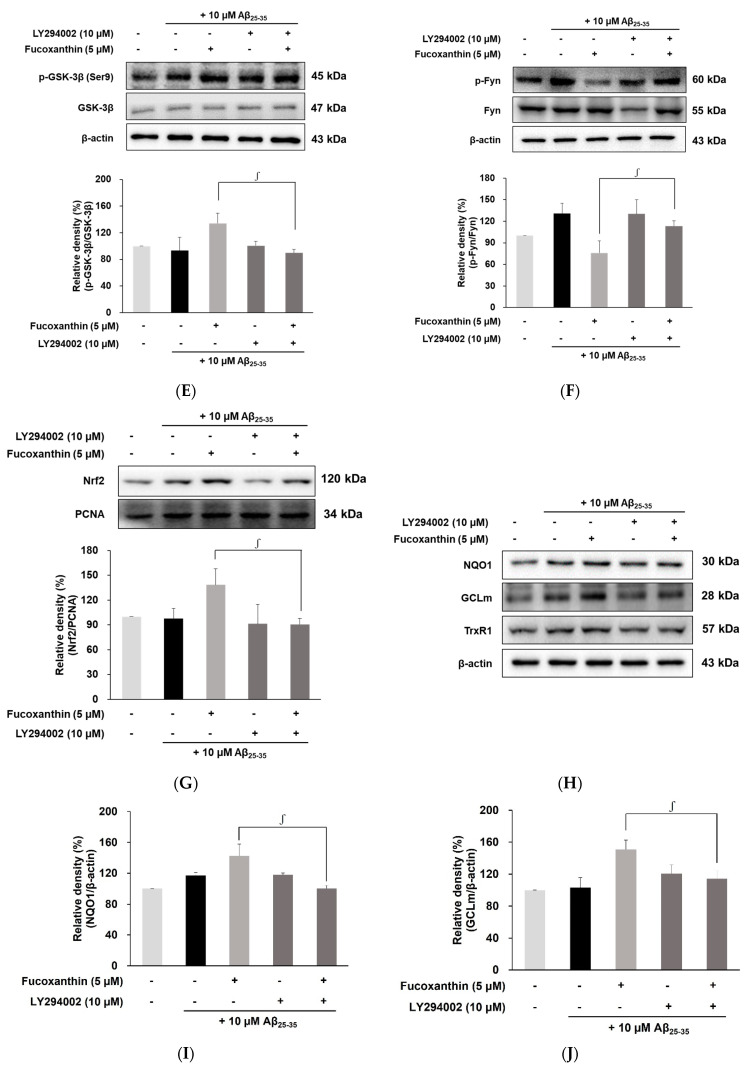

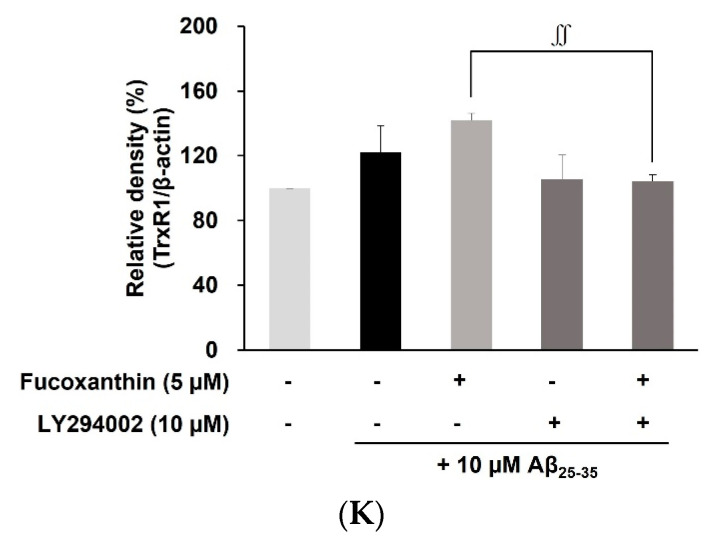
Effects of fucoxanthin on Akt/GSK-3β/Fyn signaling in Aβ_25-35_-induced PC12 cells. The cells were pretreated with the indicated concentrations of fucoxanthin for 1 h and stimulated with 10 μM Aβ_25-35_ or 1 h. The expression of (**A**) p-Akt (Ser 473)/Akt, (**B**) p-GSK-3β (Ser 9)/GSK-3β, and (**C**) p-Fyn/Fyn. Western blotting results of (**D**) p-Akt (Ser 473)/Akt, (**E**) p-GSK-3β (Ser 9)/GSK-3β, (**F**) p-Fyn/Fyn, (**G**) Nrf2, and (**H**–**K**) phase-II enzymes (NQO1, GCLm, and TrxR1) expression co-treatment of the PI3K inhibitor LY294002 and fucoxanthin. After pretreatment with the PI3K inhibitor LY294002 for 30 min, the cells were treated with 5 μM fucoxanthin for 1 h, followed by Aβ_25–35_ treatment. Results are indicated as mean ± S.D. and represent three independent experiments with 3 replications in each experiment. ## *p* < 0.01 compared with the control groups; *** *p* < 0.001 and ** *p* < 0.01 compared with the Aβ_25-35_-treated alone; §§ *p* < 0.01 compared with the Aβ_25–35_ -treated alone; ∬ *p* < 0.01 and ∫ *p* < 0.05 compared with the fucoxanthin group without LY294002.

To investigate the mechanism by which fucoxanthin promotes Nrf2 nuclear localization, the regulation of the Akt/GSK-3β/Fyn signaling pathway upon fucoxanthin pretreatment was evaluated in Aβ-induced neuronal injury. As shown in Figure 4A, fucoxanthin significantly elevated Akt phosphorylation of Ser473 compared to that of only Aβ_25-35_-treated cells. Similarly, fucoxanthin efficiently augmented the phosphorylation of GSK-3β (Ser 9), which is a downstream kinase of Akt (Figure 4B). Moreover, phosphorylation of Fyn was upregulated in cells treated with Aβ_25-35_ alone (*p* < 0.01), but fucoxanthin at 5 μM down-regulated Fyn phosphorylation to the control level, in parallel with GSK-3β inactivation and Akt activation (Figure 4C). 

To further confirm whether the Akt/GSK-3β/Fyn signaling cascade plays a critical role in Nrf2 nuclear retention by fucoxanthin, Aβ-treated PC12 cells were exposed to a LY294002, specific and potent inhibitor of PI3K/Akt, with or without fucoxanthin pretreatment. As shown in Figure 4D–F, the combination of fucoxanthin and LY294002 resulted in a significant reduction in phosphorylated Akt and GSK-3β expression, and a corresponding efficient elevation of Fyn phosphorylation compared to fucoxanthin treatment alone in Aβ_25-35_-damaged cells. Moreover, the augmented nuclear expression of Nrf2, mediated by fucoxanthin at 5 μM, was substantially lowered to the control level after co-treatment with fucoxanthin and LY294002 (Figure 4G). Finally, the combination of fucoxanthin and LY294002 reversed the effects of our compound on the expression of antioxidant enzymes, including NQO1, GCLm, and TrxR1 (Figure 4H–K). These findings suggest that fucoxanthin promotes the nuclear accumulation of Nrf2 by blocking its nuclear export through the Akt/GSK-3β/Fyn axis.

### 3.5. Molecular Docking Simulation between Fucoxanthin and Target Proteins in Antioxidant Defense System

**Figure 5:**
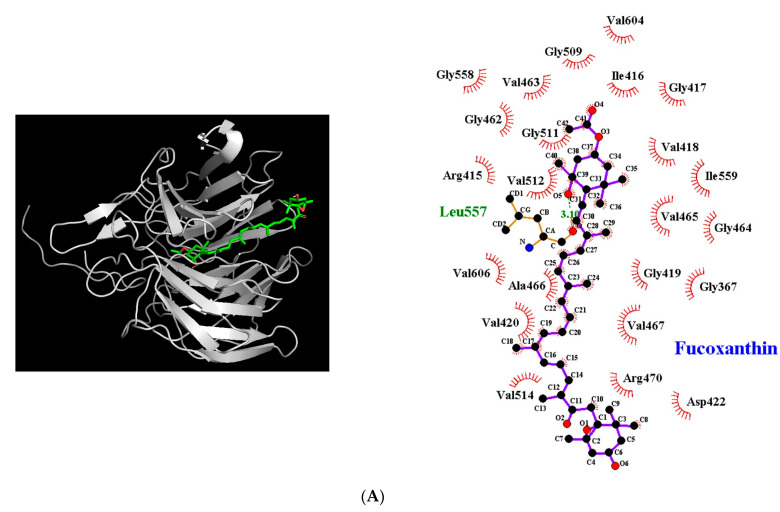

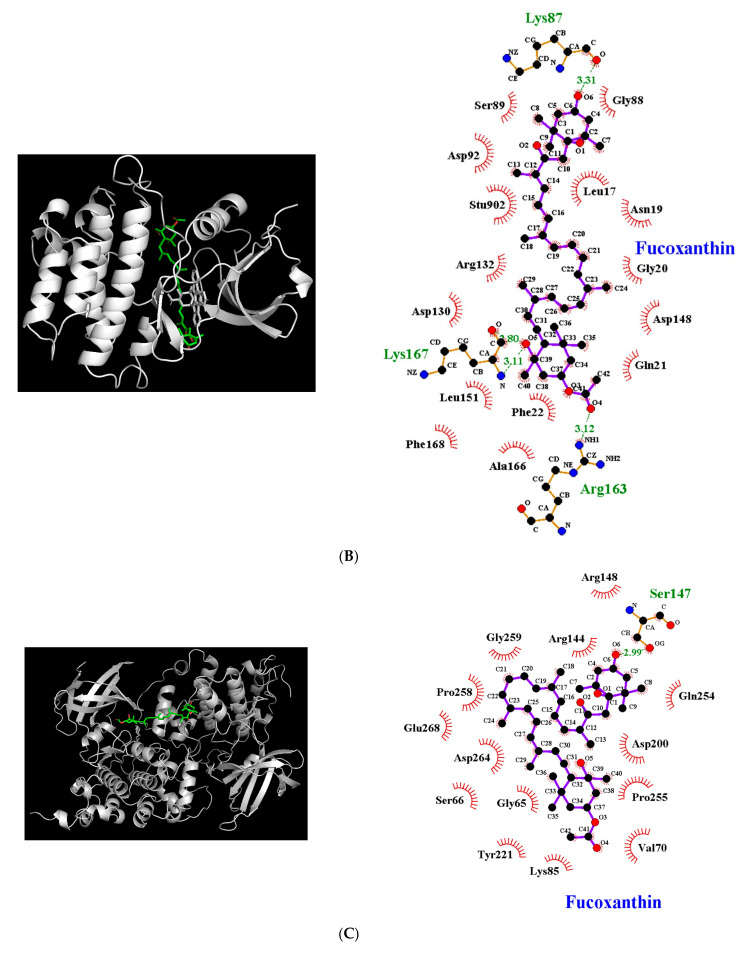

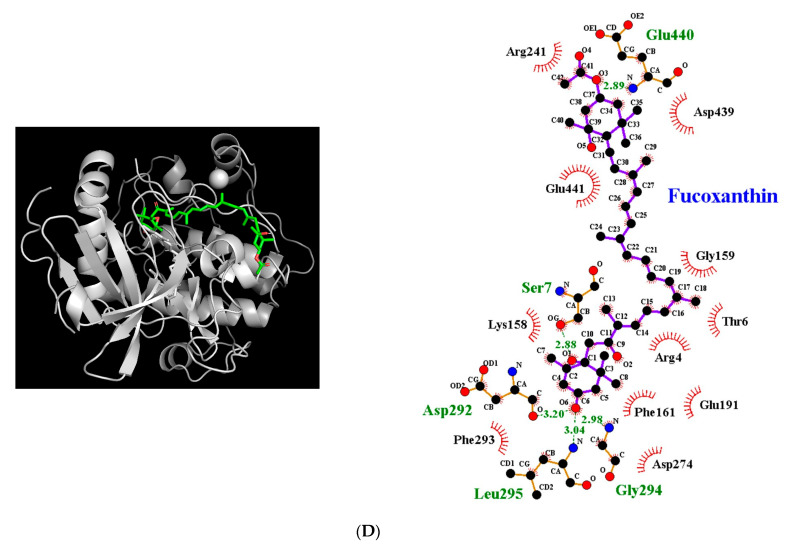
Computational docking interactions of fucoxanthin with (**A**) Nrf2-keap1 complex (**B**) Fyn, (**C**) GSK-3β, and (**D**) Akt; surface view, interaction map, and hydrogen (dotted line in green) and hydrophobic bonding (red dashed semicircle) between fucoxanthin with different targeted proteins.

Clarifying the binding interaction of fucoxanthin with Nrf2-Keap1, Fyn, GSK-3β, and Akt allows us to better understand the binding mechanism of the compound within the amino acid residues of target proteins for neuroprotective properties and to design more effective antioxidant agents. According to the results of the docking simulation (Table 2 and Figure 5A), the fucoxanthin and Nrf2-Keap 1 complex exhibited a negative binding energy (−9.4 kcal/mol). The Leu557 residue of Nrf2 with Keap 1 participated in the formation of a hydrogen-bonding interaction with 5′ the hydroxyl group of fucoxanthin, with a bonding distance of 3.1 Å. Moreover, the compound formed hydrophobic interactions with residues including Gly367, Arg415, Ile416, Gly417, Val418, Gly419, Val420, Asp422, Gly462, Val463, Gly464, Val465, Ala466, Val467, Arg470, Gly509, Gly511, Val512, Val514, Gly558, Ile559, Val604, and Val606.

The lowest binding energy of the fucoxanthin-Fyn complex was −8.1 kcal/mol (Table 2 and Figure 5B). The hydroxyl and oxygen group of fucoxanthin at C-3, C-3′, and C-5′formed four hydrogen bonds with Gly88, Arg163, and Lys167, with a bond distance of 3.31 Å, 3.12 Å, 2.80 Å, and 3.11 Å, respectively. It interacts with Fyn via van der Waals interactions with Leu17, Asn19, Gly20, Gln21, Phe22, Gly88, Ser89, Asp92, Asp130, Arg132, Asp148, Leu151, Ala166, and Phe168.

The lowest binding energy of the fucoxanthin-GSK-3β complex was predicted to be −7.4 kcal/mol (Table 2 and Figure 5C). The complex was stabilized by the formation of a hydrogen bond between the Ser147 residue and the hydroxyl group at C-3 of fucoxanthin, with a bonding distance of 2.99 Å. In addition, 14 van der Waals interactions were found between Gly65, Ser66, Val70, Lys85, Arg144, Arg148, Asp200, Tyr221, Gln254, Pro255, Pro258, Gly259, Asp264, and Glu268.

The results of docking prediction (Table 2 and Figure 5D) showed that fucoxanthin bonded with Akt and had the lowest binding energy of −8.0 kcal/mol. The five hydrogen interactions of fucoxanthin-Akt were hydroxyl group at C-3 of fucoxanthin to Asp292, Gly294, Leu295, and the oxygen group at C-5 and C-3′ of fucoxanthin to Ser4 and Glu440, with distances of 3.20 Å, 2.98 Å, 3.04 Å, 2.88 Å, and 2.89 Å. Furthermore, 11 residues of Akt, including Arg4, Thr6, Lys158, Gly159, Phe161, Glu191, Arg241, Asp274, Phe293, Asp439, and Glu441, were demonstrated to participate in van der Waals interactions with fucoxanthin.

## 4. Discussion

The Aβ acts as a direct or indirect pro-oxidant that induces oxidative stress, which represents an imbalance between ROS and the ability of the cellular defense system to detoxify reactive intermediates. Emerging evidence has suggested that antioxidants can control Aβ-induced oxidative damage as a promising strategy for AD prevention. Fucoxanthin, a xanthophyll carotenoid abundant in brown algae, has been reported to possess strong antioxidant potential. A 10 μM concentration of Aβ_25–35_ was selected based on previously reported concentrations used in vitro [18]. This study provides the first evidence that fucoxanthin exerted a neuroprotective effect and highlighted its molecular mechanisms involved in the activation of Nrf2 through the Akt/GSK-3β/Fyn signaling pathway and upregulation of the antioxidant enzymes expression against Aβ_25-35_-caused oxidative damage.

The balance between anti-apoptotic Bcl-2 and pro-apoptotic Bax proteins is an important factor in apoptosis. In the neuronal response to Aβ damage, Bax relocates from the cytoplasm to the outer mitochondrial membrane and causes mitochondrial permeability transition, resulting in an apoptotic cascade [25]. Moreover, the reduction in MMP with a simultaneous increase in the Bax/Bcl-2 expression ratio directly reflects mitochondrial-mediated apoptosis [25,26].

Accumulating evidence suggests that Aβ causes excessive Ca^2+^ release from the endoplasmic reticulum (ER) and subsequently elevates mitochondrial Ca^2+^ levels, leading to apoptosis via mitochondrial dysfunction [27,28,29]. The above results are consistent with our present study showing that exposure of neuronal cells to toxic Aβ_25-35_ obviously reduced the Bcl-2/Bax ratio and MMP, and elevated Ca^2+^ overload, which in turn triggered apoptosis. In addition, it was confirmed that the protective effect of fucoxanthin against neuronal injury by Aβ was related to regulating apoptosis associated proteins and mitochondrial function.

The Nrf2 acts as a master redox regulator that controls the inducible expression of phase II detoxification and antioxidant enzyme genes [30]. Previous studies have reported that the genetic ablation of Nrf2 markedly increases oxidative stress and inflammatory responses, whereas overexpression of Nrf2 improves spatial learning impairment and enhances neuroprotection against Aβ in an APP/presenilin 1 (PS1) transgenic mouse model [31,32,33]. Moreover, Nrf2 is predominantly observed in the cytoplasm but much less so in the nucleus in AD brains, despite the presence of oxidative stress [34]. A previous study suggested that fucoxanthin may alter the conformation of Keap1 and promote dissociation of Nrf2 from Keap1 and its subsequent nuclear translocation in 6-OHDA-expopsed PC12 cells and zebrafish [35]. The present study revealed that the level of total Nrf2 was increased without Nrf2 activation following the induction of Aβ. However, pretreatment of fucoxanthin in Aβ-induced injury in PC12 cells resulted in s substantial nuclear translocation of Nrf2, which was consistent with a reduction in Keap1 expression, suggesting that fucoxanthin upregulates Nrf2 nuclear localization by dissociation of the Nrf2-Keap1 complex.

In addition to Keap1-dependent regulation of Nrf2, GSK-3β is a critical protein involved in Nrf2 degradation and phosphorylates Nrf2 to facilitate its recognition of Nrf2 and subsequent degradation [36]. Moreover, GSK-3β indirectly phosphorylates Nrf2 via Fyn kinase and exports Nrf2 to the cytoplasm. Compelling evidence suggests that inhibition of GSK-3β activity by pharmacological treatment prevents cognitive impairment in transgenic AD mice [37]. The present results revealed that fucoxanthin inactivated GSK-3β via Akt, which induced Fyn phosphorylation, resulting in nuclear accumulation of Nrf2. To confirm that the neuroprotective activity of fucoxanthin is mediated through Nrf2, a PI3K inhibitor was employed as a specific upstream inhibitor of the Nrf2 signaling pathway. The results revealed that inhibition of the Akt/GSK-3β/Fyn signaling pathway reversed fucoxanthin-mediated expression of antioxidant enzymes and nuclear accumulation of Nrf2. Previous studies have reported that fucoxanthin exerts significant protective effects against acute kidney injury, traumatic brain injury, and skin cancer by increasing Nrf2-mediated antioxidant enzyme expression via multiple kinase pathways [38,39,40]. It has been shown that supplementation of fucoxanthin significantly enhanced expression of Nrf2 and its target gene, NQO1, in high fat diet fed rats [41].

Fucoxanthin is a major carotenoid found in brown seaweed and has a structure containing an allenic bond, epoxide group, and hydroxyl group [42]. In particular, the compound has unusual structural features, such as allene and 5,6-monoepoxide bonds, which are not found in other carotenoids in brown seaweeds [43]. Moreover, the compound has multiple oxygenic functional groups, including hydroxyl, epoxy, carbonyl, and carboxyl moieties, which contribute to its antioxidant activity [14]. According to a previous study, fucoxanthin supplementation enhanced the expression of antioxidant enzymes, including glutathione transferase and catalase, and provided more potent antioxidant activity than β-carotene [44,45]. In the present docking study, the oxygenic group of fucoxanthin at C-3, C-3′, and C-5′ participated in the formation of hydrogen bonds with Nrf-2, Fyn, GSK-3β, and Akt. Moreover, fucoxanthin acts on the targeted proteins mainly via hydrophobic interactions due to its hydrophobic chain consisting of eight conjugated double bonds and hydrophobic residues of target markers, including Gly, Pro, Val, Phe, Trp, Leu, Ile, and Ala. Especially, the present study showed that the binding site of fucoxanthin in the Keap1 pocket overlapped with Arg415, known as one of the binding sites of Nrf2. This analysis implied that the compound can compete with Nrf2 at its Keap1 kelch domain-binding site, thereby promoting nuclear translocation. Furthermore, with respect to Nrf2 activation by the upstream kinase pathway, the compound structurally binds to the Fyn, GSK-3β, and Akt proteins to exert its pharmacological activities.

The permeability of the blood–brain barrier (BBB) is an essential factor in the development of novel agents for AD prevention to exert neuroprotective effects. A previous study showed that fucoxanthin, a lipophilic pigment, penetrates the BBB and remains in the brain after oral administration (200 mg/kg), suggesting the potential of fucoxanthin to exert neuroprotective effects in the brain [16]. Regarding safety, fucoxanthin showed no signs of Ames or hepatotoxicity during in silico toxicity analysis. Beppu et al. reported no abnormality or mortality in clinical symptoms and normal organ function in Institute of Cancer Research (ICR) mice treated with fucoxanthin at a single dose of 2000 mg/kg or a repeated dose of 1000 mg/kg for 30 days [46].

## 5. Conclusions

The present findings provide scientific evidence for the first time that fucoxanthin exerts a protective effect by activating the Nrf2-mediated antioxidant system and upregulating the Akt/GSK-3β/Fyn axis during Aβ-induced oxidative stress. Moreover, molecular docking analysis demonstrated that fucoxanthin had strong interactions with Nrf2 and its upstream regulator via hydrogen bonds and van der Waals forces. The protective effect of fucoxanthin is due to the Akt/GSK-3β/Fyn-dependent Nrf2 activation. The present findings support a better understanding of the vital role of fucoxanthin in preventing AD and its potential use as a promising source of anti-AD agents.

## Figures and Tables

**Table 1:** List of primers and their characteristics.

Gene	Primer Sequences (5′→3′)	Annealing Temperature (°C)	Product Size (bp)	Genbank Accession No.
NQO1	F: ATGGCGGTGAGAAGAGCCCTG R: ACCCTTGTCATACATGGTGGC	64	408	XM_032887917
GCLm	F: AGACCGGGAACCTGCTCAAC R: CATCACCCTGATGCCTAAGC	55	1111	NM_017305
TrxR1	F: CAATGAAAAGACCGGGAAGA R: CACAGCAGCCATACTCCAAA	60	224	NM_001351984
GAPDH	F: CATCACCATCTTCCAGGAGCG R: TGACCTTGCCCACAGCCTTG	60	443	NM_017008

**Table 2:** Molecular interaction of fucoxanthin and target proteins in Nrf2 signaling pathway.

Target Protein	Binding Energy (kcal/mol)	No. of H-Bonds	H-Bonding Residues	H-Bond Length (Å)	van der Waals Residues
Nrf2-Keap1	−9.4	1	Leu557	3.10	Gly367, Arg415, Ile416, Gly417, Val418, Gly419, Val420, Asp422, Gly462, Val463, Gly464, Val465, Ala466, Val467, Arg470, Gly509, Gly511, Val512, Val514, Gly558, Ile559, Val604, Val606
Fyn	−8.1	4	Lys87 Arg163 Lys167	3.31 3.12 2.80/3.11	Leu17, Asn19, Gly20, Gln21, Phe22, Gly88, Ser89, Asp92, Asp130, Arg132, Asp148, Leu151, Ala166, Phe168
GSK-3β	−7.4	1	Ser147	2.99	Gly65, Ser66, Val70, Lys85, Arg144, Arg148, Asp200, Tyr221, Gln254, Pro255, Pro258, Gly259, Asp264, Glu268
Akt	−8.0	5	Ser7 Asp292 Gly294 Leu295 Glu440	2.88 3.20 2.98 3.04 2.89	Arg4, Thr6, Lys158, Gly159, Phe161, Glu191, Arg241, Asp274, Phe293, Asp439, Glu441

## Data Availability

The data are provided in the manuscript.
